# Brainspan: A Framework for Defining, Measuring, and Preserving Cognitive Longevity

**DOI:** 10.7759/cureus.101279

**Published:** 2026-01-11

**Authors:** Shaheen E Lakhan

**Affiliations:** 1 Bioscience, Boricua Bio, San Juan, USA; 2 Bioscience, Global Neuroscience Initiative Foundation, Miami, USA; 3 Neurology, Western University of Health Sciences, Pomona, USA; 4 Neurology, A.T. Still University School of Osteopathic Medicine in Arizona, Mesa, USA; 5 Medicine, Morehouse School of Medicine, Atlanta, USA

**Keywords:** brain aging, brainspan, cognitive longevity, cognitive resilience, healthspan, neural networks, preventive neurology, systems neuroscience

## Abstract

Longevity medicine has achieved substantial gains in extending lifespan, yet these advances have not been matched by equivalent preservation of cognitive and functional capacity. As a result, many individuals now live longer while experiencing prolonged periods of cognitive decline, emotional dysregulation, sleep disruption, and loss of independence. Existing constructs, including lifespan and healthspan, insufficiently capture the central role of brain function in determining meaningful aging outcomes. This article introduces the concept of brainspan, defined as the duration of life during which neural network efficiency remains sufficient to support autonomy, adaptive capacity, and coherent physiological and behavioral regulation. Brainspan is conceptualized as a dynamic systems property emerging from the integrated performance of cognitive, autonomic, sleep, emotional, and behavioral networks. We describe characteristic brainspan trajectories across the lifespan, identify chronic and episodic determinants of brainspan decline, discuss approaches to measuring brainspan using longitudinal, multimodal assessments, and outline implications for longevity medicine. Preserving brainspan reframes longevity from survival alone toward sustained independence, resilience, and functional agency across aging.

## Editorial

Introduction

Over the past century, medicine has achieved remarkable success in extending human lifespan. Advances in cardiovascular prevention, cancer therapy, infectious disease control, and metabolic management have dramatically reduced early mortality. However, these achievements have exposed a growing mismatch between survival and function. Increasingly, individuals live longer while experiencing extended periods of cognitive impairment, emotional instability, sleep disturbance, and declining independence.

This discrepancy reflects a limitation in how longevity has been conceptualized and measured. Longevity strategies have largely focused on preventing organ failure and reducing disease-specific mortality, often treating cognitive and behavioral decline as secondary quality-of-life issues rather than primary determinants of aging outcomes. As a result, lifespan has expanded, while the capacity for coherent thought, emotional regulation, and adaptive behavior has not kept pace.

**Figure 1:**
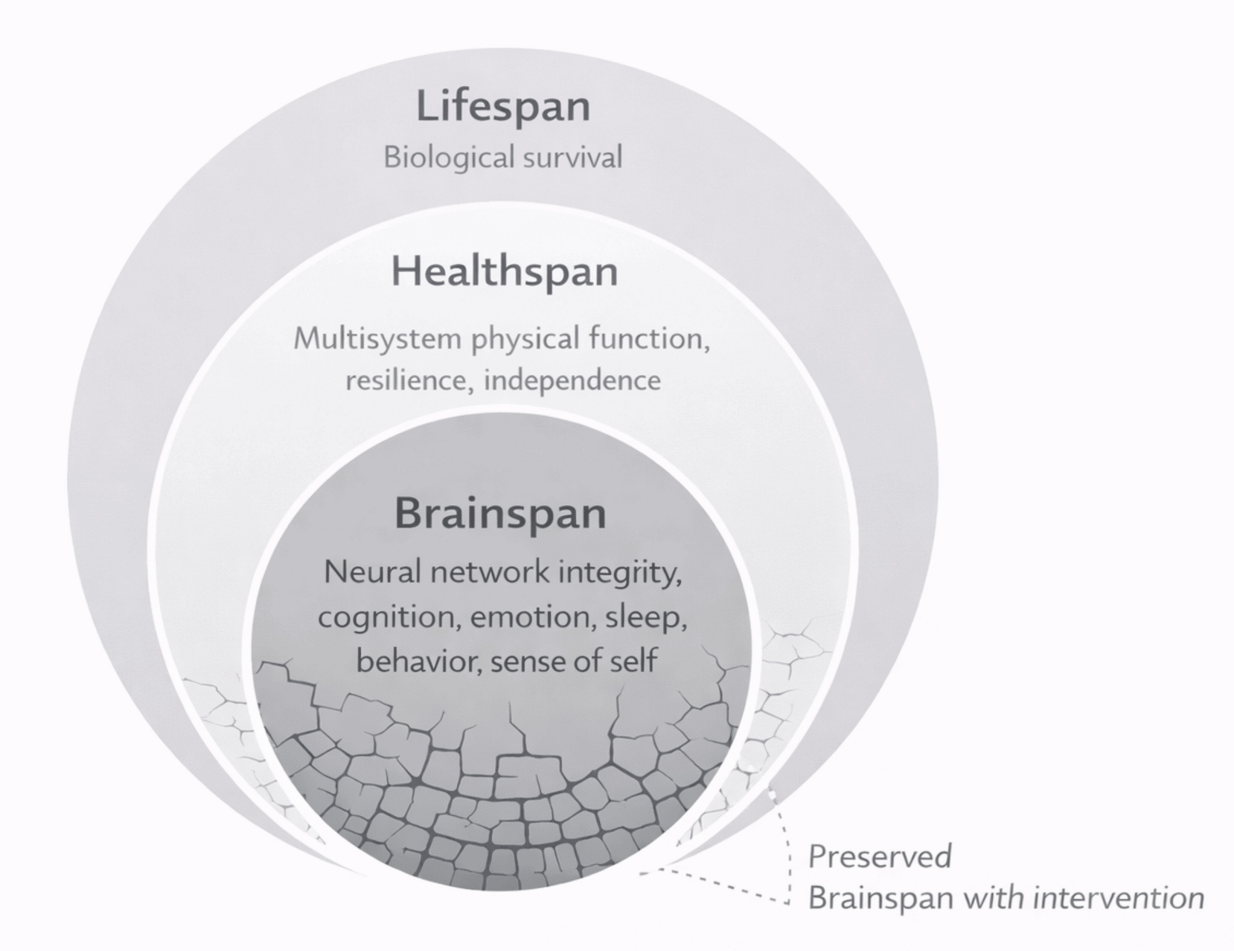
Brainspan as the foundational determinant of healthspan and lifespan This figure depicts the hierarchical relationship between brainspan, healthspan, and lifespan using a concentric systems model. Brainspan occupies the central position, representing the duration during which neural network integrity supports cognition, emotion, sleep, behavior, and sense of self. Healthspan emerges from preserved brain function and reflects multisystem physical function, resilience, and independence. Lifespan represents biological survival and can persist despite substantial loss of brainspan and healthspan. Fragmentation within the brainspan core illustrates progressive loss of neural network coherence due to aging and episodic neurotoxic stressors, which precedes and propagates outward to constrain healthspan while lifespan continues. Preservation of brainspan through intervention stabilizes downstream function, highlighting brainspan as the foundational determinant of quality of life and meaningful longevity.

The concept of healthspan emerged to address this gap by emphasizing years lived in good health rather than years lived overall (Figure 1). While healthspan represents an important advance, it remains a broad and heterogeneous construct that aggregates multiple organ systems without explicitly prioritizing the brain. Peripheral health can be preserved even as cognitive flexibility, sleep integrity, and stress tolerance deteriorate, leading to diminished autonomy and resilience despite the absence of terminal disease.

The brain occupies a unique role in aging. It integrates sensory and internal signals, regulates autonomic and endocrine function, maintains sleep and circadian rhythms, and enables adaptive behavior across changing environments. Decline in brain function undermines the organism's capacity to maintain homeostasis, respond to stress, and engage meaningfully with the world, establishing the brain as the primary coordinator of physiological resilience [1]. Recognizing this central role necessitates a construct that explicitly captures the duration of preserved brain function across the lifespan.

This article introduces the concept of brainspan as a complementary and necessary framework for understanding longevity. Brainspan focuses on the preservation of neural network efficiency and integrated brain function as the primary determinant of sustained independence, resilience, and agency during aging.

Defining brainspan

Brainspan is defined as the duration of life during which neural network efficiency remains sufficient to support autonomy, adaptive capacity, and coherent regulation of physiological and behavioral processes. Unlike lifespan, which measures survival, or healthspan, which aggregates multisystem health, brainspan centers specifically on the functional integrity of the brain as an integrated system (Table 1).

**Table 1:** Distinguishing brainspan, healthspan, and lifespan as interrelated but distinct constructs This table delineates the conceptual, biological, and clinical distinctions between brainspan, healthspan, and lifespan. While lifespan reflects biological survival and healthspan represents preserved multisystem physical function, brainspan captures the duration of preserved neural network integrity that enables cognition, behavior, emotional regulation, sleep, autonomy, and sense of self. Brainspan is positioned as the foundational determinant from which healthspan emerges and upon which meaningful longevity depends. The table highlights differences in governing constraints, early indicators of decline, measurement approaches, and therapeutic implications, illustrating why preservation of brainspan is central to durable quality of life rather than survival alone.

Domain	Brainspan	Healthspan	Lifespan
Core definition	Duration of preserved neural network integrity supporting cognition, behavior, emotion, sleep, and sense of self	Duration of preserved multisystem physical function and independence	Duration of biological survival from birth to death
Primary biological substrate	Central nervous system, distributed neural networks	Cardiovascular, metabolic, musculoskeletal, endocrine, and immune systems	Entire organism
Governing constraint	Neural network efficiency and adaptive capacity	Organ system reserve and physiological resilience	Survival despite system failure
Relationship to quality of life	Foundational determinant of agency, autonomy, meaning, and identity	Enables physical independence and function	Does not guarantee function or quality of life
Early indicators of decline	Cognitive inflexibility, sleep fragmentation, affective instability, reduced adaptability	Frailty, reduced endurance, metabolic dysregulation	None until terminal illness
Typical onset of decline	Often earliest and subclinical	Intermediate	Latest
Role of episodic events	Episodic neurotoxicity accelerates fragmentation and long-term decline	Secondary effects via downstream dysfunction	Often tolerated without immediate mortality
Measurement approaches	Cognitive performance, sleep metrics, autonomic function, network-based brain measures, behavioral adaptability	Physical performance, organ function tests, disease burden	Chronological survival
Therapeutic objective	Preserve and restore neural network coherence and adaptability	Maintain physical function and prevent organ failure	Extend survival
Consequence of neglect	Prolonged survival with loss of agency and identity	Increased disability	Extended lifespan without meaningful function
Ultimate limitation	Loss of coherent brain function	Loss of multisystem function	Death

Brainspan is not synonymous with the absence of a diagnosed neurodegenerative disease. Individuals may experience meaningful reductions in attention, executive function, emotional regulation, sleep quality, or stress tolerance long before meeting the criteria for dementia or other neurological disorders. These changes can significantly constrain independence, decision-making, and quality of life despite preserved peripheral organ function.

By focusing on network efficiency rather than isolated symptoms or diagnoses, brainspan captures subclinical and prediagnostic decline that is often invisible to traditional longevity metrics. This systems-level perspective reflects the brain's role as the coordinating organ of aging, shaping functional outcomes across domains.

Brainspan as a systems property

Brainspan emerges from the coordinated performance of multiple interacting neural systems rather than from any single cognitive or biological marker. Cognitive networks support attention, memory, executive control, and learning. Autonomic networks regulate cardiovascular stability, metabolic balance, and stress responses. Sleep and circadian systems maintain synaptic homeostasis, energy regulation, and metabolic clearance. Emotional and motivational circuits influence resilience, behavior, and social engagement.

These systems are deeply interdependent. Disruption in one domain often propagates across others, amplifying global decline. Chronic sleep fragmentation impairs cognitive performance, increases emotional reactivity, and destabilizes autonomic regulation [2]. Persistent stress dysregulation degrades executive function, sleep integrity, and immune balance. Emotional instability reduces cognitive flexibility and adaptive behavior. As a result, brainspan reflects the integrity of the system as a whole rather than the performance of any individual component.

This systems perspective explains why brain aging is frequently nonlinear. Individuals may appear stable for extended periods, followed by abrupt declines triggered by illness, psychological stress, or sleep disruption. These inflection points reflect threshold effects within interconnected networks rather than gradual neuronal loss alone.

Neurobiological substrates of brainspan

At a neurobiological level, brainspan reflects the capacity of distributed neural networks to sustain efficient information processing under conditions of metabolic constraint, environmental stress, and aging-related change. Rather than being defined by neuronal loss alone, brainspan is governed by the integrity of network-level properties, including signal-to-noise ratio, synchronization across cortical and subcortical circuits, and the energetic efficiency of neural communication [3].

As the brain ages, multiple convergent processes degrade these properties. Synaptic transmission becomes noisier, large-scale network coordination weakens, and the metabolic cost of maintaining cognitive output increases [4]. These changes impair the brain's ability to flexibly allocate resources, adapt to perturbation, and recover following stress [5]. Importantly, such network inefficiencies often precede overt structural pathology and may progress silently for years before manifesting as diagnosable disease.

Brainspan decline, therefore, reflects a reduction in neural efficiency rather than a simple accumulation of damage. From this perspective, cognitive slowing, emotional lability, sleep fragmentation, and reduced stress tolerance represent emergent consequences of diminished network coherence. The biological substrate of brainspan is not a single region or pathway, but the collective performance of interconnected neural systems operating near their adaptive limits. Preserving brainspan thus requires maintaining network integrity and efficiency across time, rather than merely preventing late-stage neurodegeneration. These network-level changes provide a mechanistic bridge between molecular aging processes and systems-level functional decline, situating brainspan at the interface of biology and lived experience.

Brainspan trajectories across the lifespan

Brainspan can follow distinct trajectories across individuals. Some maintain preserved brainspan, characterized by sustained cognitive resilience, stable sleep architecture, emotional regulation, and adaptive capacity well into later life. Others experience gradual brainspan erosion, with subtle but progressive declines across multiple domains that accumulate over decades. A third group exhibits accelerated brainspan collapse, often driven by recurrent neurological or psychiatric stressors, chronic sleep disruption, or prolonged stress dysregulation.

Episodic neurotoxic events play a critical role in shaping these trajectories. Conditions such as migraine, major depressive disorder, psychotic illness, traumatic brain injury, chronic pain syndromes, and severe sleep disorders impose acute physiological and metabolic stress on neural networks. Although symptoms may remit clinically, residual inefficiencies in network synchronization and energy utilization often persist. Over time, these inefficiencies steepen the slope of brainspan decline.

Understanding brainspan trajectories highlights the importance of early identification. Changes in trajectory often precede overt disability by many years, creating an opportunity for preventive intervention long before irreversible decline occurs.

Brainspan across the life course: windows of vulnerability and opportunity

Brainspan is shaped continuously across the life course, rather than declining exclusively in later life. Early neurodevelopment establishes the foundational architecture upon which lifelong neural resilience depends. Cognitive stimulation, sleep regularity, emotional regulation, and stress exposure during childhood and adolescence influence baseline network efficiency and adaptive capacity that persist into adulthood.

Midlife represents a critical inflection period for brainspan trajectories. During this stage, cumulative stress exposure, sleep disruption, metabolic instability, and recurrent neuropsychiatric conditions begin to exert disproportionate influence on neural networks. Although functional capacity may appear preserved, subtle declines in cognitive flexibility, emotional regulation, and recovery from stress often emerge during this period. These changes frequently go unrecognized yet play a decisive role in determining long-term brainspan outcomes.

Later life reflects the cumulative expression of earlier trajectories rather than a sudden onset of decline. Individuals who maintain preserved brainspan into older age often demonstrate greater resilience to illness, better recovery following physiological stress, and sustained autonomy despite age-related changes. Conversely, those with accelerated midlife brainspan erosion experience earlier loss of independence and reduced adaptive capacity, even when peripheral health is relatively preserved. Recognizing brainspan as a life-course construct highlights midlife as the most leverage-rich window for preventive intervention [6].

Measuring brainspan

No single biomarker can adequately capture brainspan. Instead, brainspan must be assessed as a composite, longitudinal construct reflecting integrated brain function over time. Relevant domains include cognitive performance and resilience, sleep quality and circadian stability, autonomic flexibility, emotional regulation, and behavioral adaptability [7].

A defining feature of brainspan measurement is its emphasis on longitudinal trajectories rather than static performance. Single time-point assessments, whether cognitive tests or neuroimaging-derived metrics, provide limited insight into adaptive capacity. Brainspan assessment instead prioritizes change over time, sensitivity to stressors, and the ability to recover following disruption.

Variability and resilience are, therefore, central features of brainspan measurement. Preserved brainspan is characterized not by uniformly high performance, but by stability, flexibility, and rapid recovery after perturbation. In contrast, declining brainspan manifests as increasing variability, prolonged recovery, and progressive erosion of baseline function across domains. Longitudinal monitoring of cognitive performance, sleep integrity, autonomic flexibility, and emotional regulation enables the detection of trajectory shifts that precede overt disability.

This approach reframes assessment from diagnostic labeling toward dynamic risk stratification. The goal is not to classify individuals as impaired or unimpaired, but to identify emerging patterns of decline that signal reduced adaptive reserve. Such patterns provide actionable targets for early intervention and allow brainspan to function as a clinically meaningful metric of cognitive longevity.

Determinants of brainspan decline

Brainspan decline is driven by both chronic and episodic factors. Chronic contributors include biological aging, cumulative stress exposure, sleep fragmentation, metabolic instability, physical inactivity, and reduced cognitive engagement. Episodic contributors include neurological and psychiatric events that impose acute stress on neural networks.

Neuroinflammation represents a convergent mechanism linking these diverse contributors. Recurrent activation of inflammatory signaling within the central nervous system disrupts synaptic efficiency, impairs network synchronization, and increases neural metabolic burden [8]. Although individual episodes may appear transient, their cumulative inflammatory impact progressively degrades neural network performance, creating a biological memory of systemic stress within the central nervous system [9]. This provides a biological explanation for how events occurring decades earlier can meaningfully constrain cognitive resilience and functional capacity later in life.

Preserving and extending brainspan

Brainspan is not fixed. It represents a modifiable trajectory influenced by early identification, longitudinal intervention, and sustained support of neural network function. Preserving brainspan requires a shift from reactive treatment of late-stage disease toward proactive maintenance of neural resilience across the lifespan.

Interventions aimed at preserving brainspan must prioritize network-level effects rather than isolated symptom relief. Neuroprotection alone is insufficient. Strategies must also support neuroadaptation, recovery from episodic stress, and maintenance of sleep integrity, autonomic balance, and cognitive engagement over time.

Evaluating interventions by their impact on brainspan rather than survival alone reframes success in terms of preserved independence, adaptability, and quality of life.

Implications for longevity medicine

A potential counterargument is that brainspan represents a rebranding of existing concepts in cognitive aging. However, cognitive aging is primarily descriptive, cataloging changes in performance across populations, whereas brainspan is explicitly normative and interventional. Brainspan reframes the objective of longevity medicine from characterizing decline to preserving adaptive capacity and agency over time; this shift necessitates moving beyond static population norms toward measuring intra-individual variability and real-world functional volatility [10].

Incorporating brainspan into longevity medicine aligns with emerging global health priorities that advocate for optimizing brain health as a foundational pillar of human capital and healthy aging [11]. Routine assessment of cognitive resilience, sleep quality, emotional regulation, and stress tolerance should become standard components of preventive care. Midlife represents a critical window for intervention, as early changes in brainspan trajectory often precede irreversible decline.

Longevity strategies that fail to preserve brainspan risk extending lifespan without preserving agency or meaning, resulting in prolonged morbidity. In contrast, interventions that stabilize or extend brainspan may yield disproportionate benefits across multiple domains of health, even without dramatic effects on peripheral biomarkers.

Brainspan provides a unifying framework that bridges neurology, psychiatry, geriatrics, and preventive medicine, aligning longevity goals with functional outcomes that matter most to individuals and society.

Unlike traditional cognitive aging frameworks, brainspan integrates cognition, emotion, sleep, autonomic regulation, and behavior into a unified systems construct. It shifts emphasis from late-stage disease to early trajectory modification and from static deficits to dynamic resilience. By doing so, brainspan provides a framework that aligns measurement, prevention, and intervention around outcomes that matter most to individuals, namely, autonomy, identity, and meaningful engagement with life.

Conclusion

Longevity is not defined solely by how long individuals live, but by how long they retain the capacity for coherent thought, emotional stability, and adaptive engagement with the world. Brainspan captures this dimension by focusing on the duration of preserved brain function across the lifespan. Preserving brainspan reframes longevity as sustained independence, resilience, and agency rather than survival alone. As longevity medicine continues to evolve, brainspan should be recognized as a central metric and primary objective for extending meaningful life across aging.
